# Supplementary material for: Novel Driver Strength Index highlights important cancer genes in TCGA PanCanAtlas patients
Source: PeerJ. 2022 Aug 11;10:e13860. doi: 10.7717/peerj.13860 (PMC9375969; doi:10.7717/peerj.13860)
Supplement: Supplemental Information 2 [file peerj-10-13860-s002.zip › COHORTS/genes plots/cohorts/2021_8_16_14_9_distribution_cohorts_females_MESO.pdf]

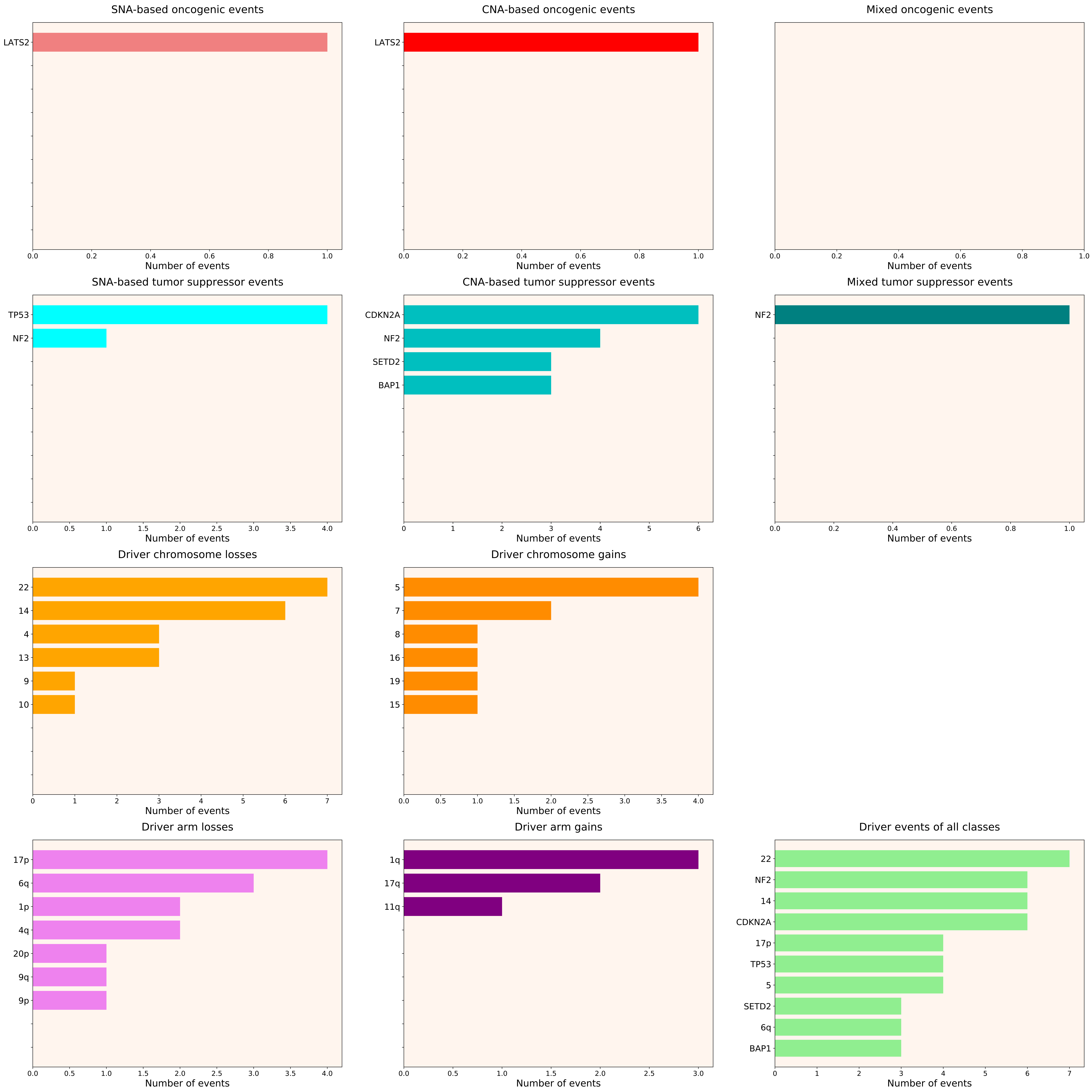

SNA-based oncogenic events
LATS2
CNA-based oncogenic events
LATS2
Mixed oncogenic events
SNA-based tumor suppressor events
TP53
NF2
CNA-based tumor suppressor events
CDKN2A
NF2
SETD2
BAP1
Mixed tumor suppressor events
NF2
Driver chromosome losses
22
14
4
13
9
10
Driver chromosome gains
5
7
8
16
19
15
Driver arm losses
17p
6q
1p
4q
20p
9q
9p
Driver arm gains
1q
17q
11q
Driver events of all classes
22
NF2
14
CDKN2A
17p
TP53
5
SETD2
6q
BAP1
Number of events
